# Correlation of Suicidal Thoughts and Toxoplasmosis in Patients With Depression

**DOI:** 10.7759/cureus.13369

**Published:** 2021-02-16

**Authors:** Ilkay Bahceci, Bulent Bahceci, Senol Senturk, Ilknur E Yildiz, Zihni A Yazici

**Affiliations:** 1 Medical Microbiology, Recep Tayyip Erdoğan University, Medical Faculty, Rize, TUR; 2 Psychiatry, Recep Tayyip Erdoğan University, Medical Faculty, Rize, TUR; 3 Gynecology and Obstetrics, Recep Tayyip Erdoğan University, Medical Faculty, Rize, TUR; 4 Infectious Diseases, Recep Tayyip Erdoğan University, Medical Faculty, Rize, TUR

**Keywords:** depression, depressive patients, anti-toxoplasma gondii igg, suicidal thoughts

## Abstract

Objective

We investigated the correlation between serum anti-*Toxplasma gondii* IgG and suicidal thoughts in depressive patients.

Methods

Depressive patients with (n = 100) and without (n = 100) suicidal thoughts along with 100 healthy control subjects were recruited for this study. In all three groups, a semi-structured clinical interview form called Structured Clinical Interview for DSM-IV (Diagnostic and Statistical Manual of Mental Disorders) Axis-I Disorder (SCID-I), Hamilton Depression Rating Scale (HAMD), suicidal behavior scale, and a sociodemographic data form were completed. Sera from all participants were taken, and anti-toxoplasma IgG was measured by Enzyme Linked Immunosorbent Assay (ELISA)-Chemiluminescent Microparticle Immunoassay. Statistical analysis of the data was performed.

Results

The serum anti-toxoplasma IgG levels of patients with suicidal thoughts were significantly higher than those without suicidal thoughts and the controls, which were 80.04 ± 40.66, 78 ± 14.82, and 19.98 ± 14.65, respectively, p < 0.001. There was no correlation between toxoplasma IgG and HAMD score in patients lacking suicidal thoughts (r = -0.112, p = 0.463).

Conclusion

This study shows a correlation between seropositivity for anti-*Toxoplasma gondii* IgG and depression with suicidal thoughts.

## Introduction

Toxoplasmosis and depression are close concepts in their stages in modern, isolated human life. Feline contact by humans is on the increase. In a report prepared by the World Health Organization (WHO) on World Mental Day (October 10, 2012), whose main theme was "Depression as a Global Crisis," depression accounted for 4.3% of the global illness burden and was the third leading cause of illness [1]. It has been reported that 20% of the emerging diseases due to psychiatric disorders in Europe are resulted from depression, and in some countries this rises up to 26%. Lifetime risk for major depressive disorder was 5%-12% in men and 10%-25% in women, and the point prevalence in community samples of major depressive disorder in adults ranged from 5%-9% for women to 2%-3% for men. Depression is an important public health problem that causes 3000 deaths per day as a result of suicide. Longitudinal follow-up studies show that 10%-15% of suicide attempts have been successful [1,2].

*Toxoplasma gondii* is a zoonotic intracellular parasite that is reported to have infected approximately one third of the world population [3]. The sexual reproduction stage of this protozoan is carried out in the small intestinal epithelium of felines, and the excreted oocysts are taken by humans and other mammals coincidentally with water and food. Mammalian infections occur more often with the sporozoites that develop in the oocyst [4]. *T. gondii *oocysts that are resistant to gastric acid infect all cells in the digestive tract, then cross the blood-brain barrier (BBB), settle in astrocytes and neurons, and remain latent in the brain for a long time [5].

Even though the details of the mechanism of crossing the BBB are unclear, the parasite is known to localize in the orbitofrontal cortex, amygdala, hyppocampus, anterior cingulate cortex, and basal ganglion as revealed by neurological scanning for depression [6-8]. *T. gondii* can cause depressive behavioral changes such as nervousness and aggression, which are risk factors for suicide attempts by causing changes in host serotonergic systems [9-12]. In previous studies, although the relationship between toxoplasma seropositivity and suicide attempt was investigated in depressed patients, no study investigating the relationship between this parasite and suicidal ideation was found, which is the focus of this study.

## Materials and methods

Hundred depressive patients (aged 18-65 years) with suicidal ideation (group I), 100 depressed patients without suicidal ideation (group II), and 100 healthy control subjects (group III) were recruited for the study. The patients were selected from those who remotely consulted the hospital psychiatry outpatient clinic. Informed consent was obtained from all three groups. Exclusion criteria included pregnancy; psychiatric and any other drug treatment; endocrinological, metabolic, neurological, cardiologic, infectious disease; additional psychiatric comorbidity except for depression; and psychiatric disease in the control group. In all three groups, a semi-structured clinical interview form called Structured Clinical Interview for DSM-IV (Diagnostic and Statistical Manual of Mental Disorders) Axis-I Disorder (SCID-I) [13], a Hamilton Depression Rating Scale [14], a suicidal behavior scale [15], and a sociodemographic data form drawn by the investigators were completed for DSM-IV Axis-I diagnoses.

From the antecubital region of all participants, 2 ml of venous blood was taken and centrifuged at 4000 rpm for 10 minutes, and the serum was stored at -20°C. Sera from all participants were screened/titrated by an Enzyme Linked Immunosorbent Assay (ELISA) kit provided by Architect System, Abbott Diagnostics, Germany, in accordance with the criteria of macro-ELISA and chitin evaluation as per recommendation of the manufacturer. Anti-toxo IgG values that ​​ranged from zero to 1.6 were considered non-reactive, while those with values greater than 1.6 ​​were considered reactive. Ethical approval was obtained from the Medical School Clinical Studies Ethics Committee (Decision No: 2014/130).

Statistical evaluation

Statistical analysis was performed using the Statistical Package for Social Sciences (SPSS) version 18.0 (IBM Corp., Armonk, NY). Normal distribution of continuous variables was tested with Kolmogorov-Smirnov test. Chi-square test was used for categorical variables, and student's t-test was used for continuous variables. Correlation analysis was performed by the Spearman correlation test. One-way ANOVA with Dunnett’s post-hoc test was used. The level of significance was set as p < 0.05.

## Results

There were no significant differences among the groups for gender distribution (p = 0.105) and mean age (p = 0.107) (Table 1). Hamilton depression scores, the presence of anti-toxoplasma IgG, and the IgG levels of the groups are shown in Table 1. 

**Table 1:** Data for depressive patients with (I) and without (II) suicidal ideation and healthy control (III) groups Group I: depressive patients with suicidal ideation, group II: depressive patients without suicide ideation, group III: healthy control. ^a^Chi-square test, ^b^Student's t-test, ^c^One-way ANOVA test post-hoc Dunnett test. SD: Standard deviation; HAMD: Hamilton Depression Rating Scale. p < 0.05: Statistically significant.

Parameters		Group I	Group II	Group III	p Value
						Gender					
Female	n (%)	65 (65)	68 (68)	61 (61%)	0.105^a^
Male	n (%)	35 (35)	32 (32)	39 (39%)	
Age	Mean ± SD	30.14 ± 7.29	31.94 ± 8.19	29.73 ± 8.43	0.107^b^
Toxo IgG					
Positive (n)	n (%)	37 (37)	35 (35)	21 (21)	<0.001^a^
Negative (n)	n (%)	63 (63)	65 (65)	79 (79)	
HAMD	Mean ± SD	23.14 ± 6.24	20.90 ± 3.34		<0.001^c^
	Mean ± SD	23.14 ± 6.24		3.76 ± 1.61	<0.001^c^
	Mean ± SD		20.90 ± 3.34	3.76 ± 1.61	<0.001^c^
Toxo IgG level	Mean ± SD	80.04 ± 40.66	36.78 ± 14.82	19.98 ± 14.65	<0.001^c^

While there was a significant correlation between the HAMD scores and IgG values for group I ​​(r = 0.650; p < 0.001), this was not the case for group II (r = -0.112; p = 0.463). No correlation was found between these two groups in terms of age and gender (Table 2).

**Table 2:** Correlation of HAMD scores and IgG values Spearman correlation test, p < 0.05. HAMD: Hamilton Depression Rating Scale.

IgG	Group I		Group II	
	r	p	r	p
HAMD	0.65	< 0.001	-0112	0.463
Age	0.005	0.966	0.137	0.103
Gender	0.187	0.126	-0.129	0.399

## Discussion

Our study differs from previous studies in terms of investigating the relationship between suicide and *T. gondii* IgG seropositivity. Incorporation of depressive patients who did not attempt suicide in the study but had suicidal ideation during the study accounts for this difference. There has been no other study utilizing a similar methodology to study the relationship between HAMD scores and suicidal ideation in the literature.

In recent years, rapidly increasing suicide rates proved to be an important health problem for many countries, including Turkey. Therefore, clinicians must be aware of the assessment methods of the risk factors to enable them to take preventive/deterring measures [16,17]. For this reason, a test that can be easily accessed, cheap, fast, and accurate can be drawn by investigators for predicting possible suicide.

Seropositivity for *T. gondii* IgG varies worldwide and ranges between 15% and 87% [18]. In Turkey, this rate was found to be 23.1%, which is similar to our findings (21%) for group III [19]. Seropositivity for *T. gondii* IgG was found to be between 13% and 41% in previous studies in depressive patients [3,20,21]. In this study, this positivity was 37% and 35% for depressive patient groups I and II, both with and without suicidal ideation, respectively. These differences in studies are thought to be related to factors such as nutritional habits, severity of climate, and pet care.

The relationship between seropositivity for *T. gondii* IgG and suicide attempt has been investigated. The seropositivity rates of individuals who attempted suicide were found to be higher than those of the control group [19,20,22-25]. Unlike these studies and our study, Alvarado-Esquivel et al. found no relationship between *T. gondii* IgG seropositivity and suicide [26].

However, this study shows that there is a strong correlation between the HAMD scores of depressive patients who are suicidal and contemplating suicide. No correlation was found between the HAMD scores of depressive patients without suicidal thoughts and the suicidal ideation group. In addition, IgG levels in patients with and without suicidal ideation were statistically significantly different and higher in patients with suicidal ideation. These results suggest that *T. gondii *Ig G levels may be important in predicting suicide, an important public health problem, and that our research will be the precursor to further work.

The limitations of this study include low subject numbers and confinement in the same geographic region.

## Conclusions

This study differs from previous studies in terms of investigating the relationship between suicide and *T. gondii* IgG seropositivity. Incorporation of depressive patients who did not attempt suicide in the study but had suicidal ideation during the study accounts for this difference. There has been no other study utilizing a similar methodology to study the relationship between HAMD scores and suicidal ideation in the literature. The data shows that the seropositivity of *T. gondii* IgG in depressive patients with or without suicidal ideation is higher than the healthy control and that it is even higher in patients with suicidal ideation than those without.
